# The impact of the COVID-19 pandemic on undergraduate and postgraduate students: A cross-sectional survey

**DOI:** 10.3389/fpsyt.2023.1074597

**Published:** 2023-02-03

**Authors:** Lu Zhu, Ying Zhou, Yiyue Huang, Xinxin Lei, Haoran Guo, Yibo Hu, Songjiang Wu, Li Lei, Aiyuan Guo

**Affiliations:** ^1^Department of Dermatology, Third Xiangya Hospital, Central South University, Changsha, China; ^2^Department of Hepatobiliary Surgery, Affiliated Tumor Hospital of Guangxi Medical University, Nanning, China

**Keywords:** COVID-19, physical and mental health, daily life, learning situation, graduation, undergraduate and postgraduate students

## Abstract

**Background:**

The COVID-19 pandemic has impacted many facets of life. This study focuses on undergraduate and postgraduate students in China to explore how the pandemic has affected health status, daily life, learning situations, graduation-related situations, and their studies or work planning.

**Methods:**

This study sent online questionnaires to 2,395 participants to investigate the extent to which they were affected by the epidemic in the various aspects mentioned above and to understand what help they tend to get in the face of these effects.

**Results:**

A total of 2,000 valid questionnaires were collected. The physical health of 82.90% of the respondents was affected to varying degrees, with male students, non-medical students, and graduates being more affected than female students, students with medical majors, and non-graduates, respectively. The proportion of students affected by mental health, the total amount of physical exercise, emotional life, and interpersonal communication was 86.35, 88.65, 80.15, and 90.15%, respectively. Compared with medical students and non-graduates, non-medical students and graduates were more affected. In addition, students’ learning and graduation conditions have also been affected to a certain extent: 13.07% of students may not be able to graduate on time, and the proportion of postgraduate students’ graduations affected was higher than that of undergraduate students.

**Conclusion:**

The COVID-19 pandemic has affected the health status of students, their daily lives, learning situations, and so on to varying degrees. We need to pay attention to the issues, provide practical solutions, and provide a basis for better responses to similar epidemics in the future.

## 1. Introduction

Since the outbreak of the Coronavirus disease 2019 (COVID-19) epidemic, many countries have implemented relatively strict blockades or curfews, such as regional blockades and mask wearing. Since January 2021, China’s domestic epidemic situation has been effectively controlled, adhering to the general strategy of “guarding against imported cases and preventing a resurgence of the outbreak at home,” and carrying out “centralized isolation” of close contacts. On December 7, 2021, China formally put forward the “dynamic zero-COVID policy,” which is to take effective and comprehensive measures when a local case occurs under the premise of “one case found, one case cured” (1), these control measures have gradually become the norm, and people in this environment have been affected in many ways.

As a densely populated area, schools have suspended offline teaching activities. In view of the prevalence of the epidemic and the obstacles to academic progress, the psychological state of many students has also changed to various degrees. A previous study surveyed 612 college students at the University of California, Los Angeles, and found that they were prone to psychological problems such as depression, anxiety, and stress during the pandemic (2). Anxiety symptoms were positively correlated with adverse effects on daily life and delays in academic activities, but the level of anxiety was negatively related to social support (3). Interestingly, some studies have found differences in the psychological state of medical and non-medical students affected by the outbreak. One study found that medical students were exposed to greater pressure and negative emotions than non-medical students because of their familiarity with the COVID-19 epidemic (4). However, medical students refuse to seek psychological help due to professional stigma (5), distance constraints, and the costs of counseling services (6). Another study reported the contrary finding. When they examined the immediate psychological effects of medical students and non-medical students, they found that medical students’ assessment of the epidemic was more severe and catastrophic than that of non-medical students, but also that medical students reported fewer mental health problems than non-medical students, showing that accurate and transparent information about the epidemic is helpful for mental health (7).

To avoid interfering with student learning processes, many schools have introduced e-learning, primarily to provide distance learning for students in the form of recorded classes or online meetings. A study found that 62.2% of 456 medical students were more satisfied with online courses than offline courses (8), and the prevalence of depression and burnout syndrome decreased after the transition from traditional to online learning, but this transition had a negative impact on students’ communication and interpersonal relationships (9). Additionally, most of the students surveyed opposed the use of webcams in the classroom for privacy reasons (10), which suggests that we should also pay attention to protecting students’ privacy when conducting online education.

The impact of the normalized prevention and control of the epidemic has penetrated all aspects of students’ lives, and it is far from sufficient to focus on the above problems. Today’s college students are facing an increasing number of problems, such as food insecurity (11), lack of belonging (12), uncertainty about the future (13), etc.; and the COVID-19 epidemic is likely to exacerbate these issues. Currently, there is a lack of research for undergraduate and postgraduate students. Hence, this survey aims to conduct a cross-sectional study on the above aspects to understand the extent to which the epidemic has affected students in these areas, to provide some measures for solving these problems, and to put forth certain theories to find solutions to the difficulties that students face during similar pandemic events.

## 2. Participants and methods

### 2.1. Participants

An online survey was sent to Chinese undergraduate, master’s and doctorate students between March 12, 2021 and April 12, 2021. Participants provided written informed consent (see Supplementary Text 1) to participate in the study, which they read before completing the online questionnaire. If they accepted this notification, they would continue to respond to the survey, otherwise the survey was terminated. In addition, this survey is anonymous, the information that identifies individual participants will not be obtained during or after data collection, and the original survey information will not be disclosed.

### 2.2. Questionnaire contents

In this study, an online survey was conducted using a self-created questionnaire. This questionnaire consists of four parts: (1) the basic demographic information of the respondents, which includes gender, age, academic degree, major, graduation situation, and the risk level of the epidemic at their school over the past 3 months (Selecting the highest risk level for the school location during this period); (2) the effect of the epidemic on respondents in the following areas: the health status of undergraduate and postgraduate students (including physical health, mental health, and the total amount of physical exercise), students’ daily life (including leisure and entertainment activities, emotional life, and interpersonal communication), and the students’ learning situations (including learning style, learning time, and learning efficiency) were each rated by the respondents on a scale from 0 (no impact at all) to 10 (very large impact); (3) the specific changes in physical health, daily life and learning situations of the respondents during the COVID-19 epidemic were rated with a score of 1–5; and (4) the respondents’ graduation-related situations (including internship and research topic) and the degree of impact those items had on their further study or work planning.

### 2.3. Data collection

The survey was entered and approved *via* the Questionnaire Star platform. Before the questionnaire was officially distributed, a preliminary investigation was conducted and the content of the questionnaire was adjusted to reflect respondent feedback. When collecting the questionnaire, we restricted access to the device’s IP address to ensure that each respondent completed the questionnaire only once; additionally, we filtered out questionnaires with obvious errors.

### 2.4. Statistical analysis

IBM Spss Statistics 22 software was used to analyze the data, while GraphPad Prism 8.0 and R-4.1.1 were used to draw the associated graphics. In this questionnaire, measurement data are expressed as an Median (M) and Inter-Quartile Range (IQR) and qualitative data are expressed as frequencies or percentages. Differences among students with different demographic backgrounds affected by the epidemic were analyzed using the Mann–Whitney U test, the Kruskal–Wallis H test, or the chi-square test. Multiple-choice questions were analyzed through a multiple-answer analysis. In this study, the variance was statistically significant at *P* < 0.05 (*P* = 0.05).

## 3. Results

### 3.1. Basic information of the participants

A total of 2,395 online questionnaires were collected, and 2,000 valid questionnaires were analyzed after excluding 379 questionnaires from non-students and 16 incorrect questionnaires, resulting in a valid rate of 83.51%. Participants’ basic demographic information is presented in Table 1.

**TABLE 1:** The basic information of the participants (*N* = 2,000).

Variables		Number	Proportion (%)
Gender	Male	681	34.1
	Female	1,319	66.0
Age (year)	<18	11	0.5
	18–21	997	49.9
	22–25	842	42.1
	26–29	106	5.3
	≥30	44	2.2
Academic degree	Undergraduate	1,543	77.1
	Postgraduate	392	19.6
	Doctors	65	3.3
Major	Medical	963	48.2
	Non-medical	1,037	51.8
Whether a graduate	Yes	574	28.7
	No	1,426	71.3
The risk level of the epidemic at the school location	High-risk area	11	0.5
	Medium-risk area	31	1.6
	Low-risk area	1,958	97.9

### 3.2. The impact of the COVID-19 epidemic on physical health, mental health, and the total amount of physical exercise

**FIGURE 1:**
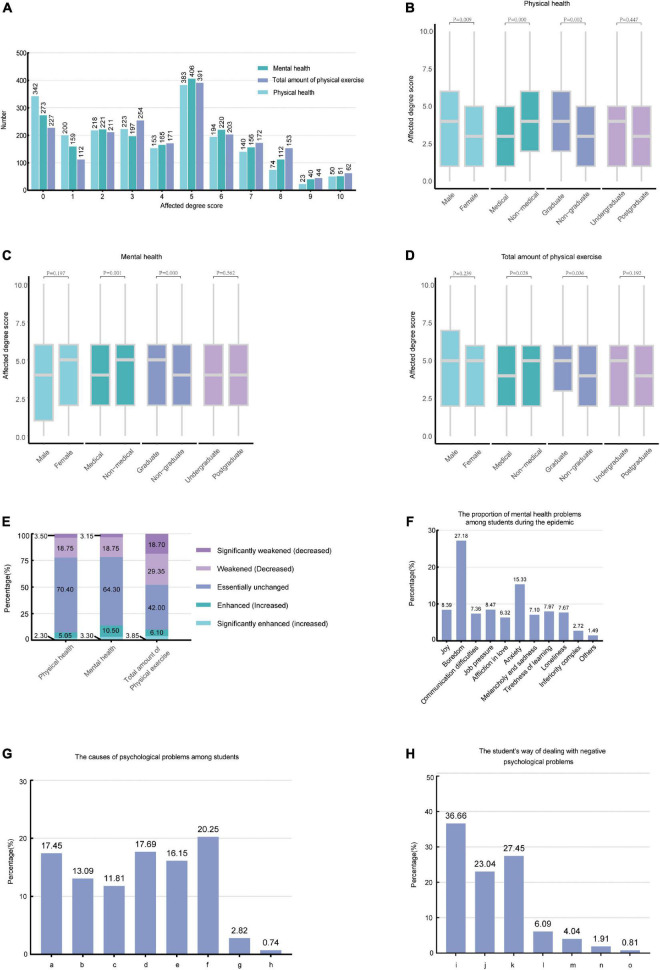
The impact of the COVID-19 epidemic on physical health, mental health, and amount of physical exercise **(A)** The frequency distribution of the scores for the impact of the pandemic on physical health, mental health, and amount of physical exercise in undergraduate and postgraduate students (0–10 points). **(B–D)** The Mann–Whitney U Test results of the health status of students with different demographic backgrounds affected by the COVID-19 epidemic. **(E)** Changes in the physical health, mental health, and the total amount of physical exercise of undergraduate and postgraduate students during the COVID-19 epidemic. **(F)** The proportion of mental health problems among students during the epidemic. **(G)** The causes of psychological problems among students. [a: There were many inconveniences in life (e.g., it is inconvenient to buy everyday necessities). b: Worried about the control of the epidemic. c: Cannot be with friends, family or lovers. d: The COVID-19 epidemic had an impact on my academic progress. e: The epidemic increased the uncertainty about future work or further studies. f: Spending too much time at home during the epidemic. g: Non-epidemic effects. h: Others]. **(H)** The student’s way of dealing with negative psychological problems. (i: Self-adjustment. j: Communicating with friends, family, etc. k: Easing my mood through physical exercise and listening to music. l: Using diary alleviation. m: Helping myself to properly understand these negative emotions by attending psychological lectures, courses, etc. n: Seeking help from a professional psychological counselor. o: Others).

In this survey, the frequency distribution of scores for the impact of the COVID-19 epidemic on physical health, mental health, and the total amount of physical exercise of undergraduate and postgraduate students are shown in Figure 1A; the higher the score, the greater the impact. By using the Mann–Whitney U Test (see Supplementary Table 1) and the Kruskal–Wallis H Test (see Supplementary Table 2), we found that: (1) males, non-medical students, and graduates were more affected in terms of physical health than females, medical students, and non-graduates, respectively, but there was no statistically significant difference between undergraduate and postgraduate students (*P* > 0.05) (Figure 1B); and (2) non-medical students and graduates were more affected in terms of mental health and the total amount of physical exercise than medical students and non-graduates, respectively (Figures 1C, D). In addition, the results of the statistical analysis of the extent to which the health status of students in different risk-level areas has been described in Supplementary Text 2.

Five levels of health status were presented to and completed by the participants to clarify how students’ physical and mental health changed due to the COVID-19 epidemic. The percentages of students who indicated each level with regard to their change in physical and mental health were shown in Figure 1E. Regarding the mental state of undergraduate and postgraduate students during the COVID-19 epidemic, the results of the multiple response analysis showed that boredom (response rate = 27.18%) and anxiety (response rate = 15.33%) were the main mental health problems. The remaining reported issues are shown in Figure 1F.

To determine the respondents’ reasons for their negative mental health states (all other than joy), we listed seven possible reasons for them to choose. The response rates were as follows: spending too much time at home during the epidemic (20.25%); the COVID-19 epidemic had an impact on their academic progress (17.69%); for other reasons, please see Figure 1G. And to understand how students deal with these psychological problems, we have created corresponding survey content. The results showed that 36.66% of students tended to self-adjust, 27.45% tended to ease their mood through physical exercise and listening to music, and only 1.91% of students chose to seek help from a professional psychological counselor (Figure 1H).

### 3.3. The impact of the COVID-19 epidemic on daily life

**FIGURE 2:**
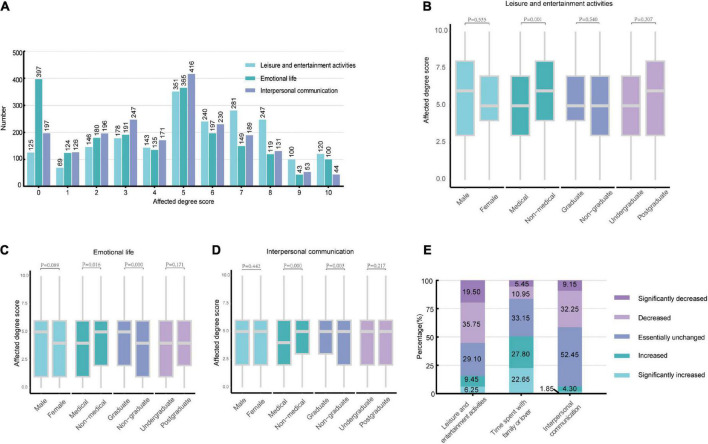
The impact of the COVID-19 epidemic on daily life **(A)** The frequency distribution of the scores for the pandemic on the daily lives of undergraduate and postgraduate students (0–10 points). **(B–D)** The Mann–Whitney U Test results of the daily life of students with different demographic backgrounds affected by the COVID-19 epidemic. **(E)** Changes in the daily lives of undergraduate and postgraduate students during the COVID-19 epidemic.

We also investigated the impact of the epidemic on the daily lives of undergraduate and postgraduate students. The frequency distributions of the impact scores are shown in Figure 2A. Through the Mann–Whitney U Test (see Supplementary Table 3) and the Kruskal–Wallis H Test (see Supplementary Table 4), we found that non-medical students’ leisure and entertainment activities were more affected by epidemics than those of medical students (Figure 2B). In terms of emotional life and interpersonal communication, non-medical students were more affected than medical students, and graduates were more affected than non-graduates, but there was no statistically significant difference between students of different genders, undergraduate students versus postgraduate students (Figures 2C, D). And the results of the statistical analysis of the extent to which daily life in different risk-level areas was affected by the epidemic were described in Supplementary Text 3. In addition, 55.25% of students reported that their leisure and entertainment activities decreased or significantly decreased during the COVID-19 epidemic compared with those of the previous period, and 50.45% of students reported that their time with family or lovers increased or significantly increased during the epidemic, while 52.45% of students reported that their interpersonal activities were essentially unchanged (Figure 2E).

### 3.4. The impact of the COVID-19 epidemic on learning situations

**FIGURE 3:**
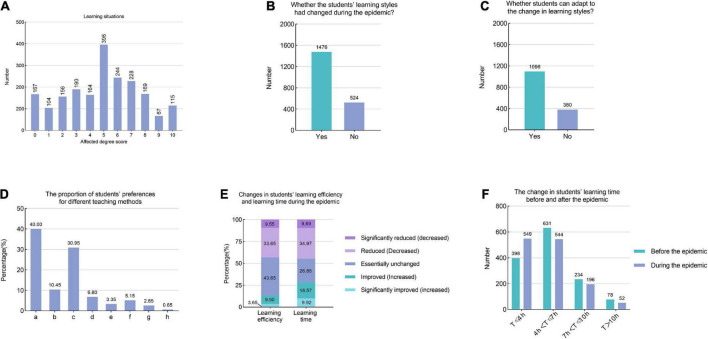
The impact of the COVID-19 epidemic on learning situations **(A)** The frequency distribution of the scores for the pandemic on learning situations in undergraduate and postgraduate students (0–10 points). **(B)** Whether the students’ learning styles had changed during the epidemic? **(C)** Whether students can adapt to the change in learning styles? **(D)** The proportion of students’ preferences for different teaching methods. (a: Offline teaching. b: Interactive online lessons. c: Online and offline hybrid teaching. d: Self-learning through the recording of videos and course materials from the teachers. e: Independent learning through Massive Open Online Course (MOOC), rain classes, wisdom trees, and other online class platforms. f: Self-study by reading the paper version of the textbook. g: Problem-based Learning. h: Others). **(E)** Changes in students’ learning efficiency and learning time during the pandemic. **(F)** The change in students’ learning time before and after the epidemic.

This study also examined how the learning situations of undergraduate and postgraduate students were affected by the pandemic (Figure 3A). Statistical analysis showed that there was no significant difference between groups as to the degree of impact of the epidemic on student learning situations (all *P* > 0.05). A total of 73.80% of the students’ learning styles changed during the epidemic (Figure 3B), and 25.75% of the students could not adapt to the change in learning styles (Figure 3C). Forty percent of the students preferred offline teaching, 30.95% preferred online and offline hybrid teaching, and the preferred proportions for other learning methods are shown in Figure 3D.

Students’ learning efficiency and study time also changed to some extent after the epidemic began (Figure 3E). Before the outbreak of the epidemic, the percentages of students’ learning time (expressed as “T”) were 4 h < T ≤ 7 h (46.10%), T ≤ 4 h (31.55%), 7 h < T ≤ 10 h (17.05%), and T > 10 h (5.30%). During the epidemic, 67.05% of the students reported that their learning time had changed, and the proportion of the above-mentioned learning time was 40.57, 40.94, 14.62, and 3.88%, respectively (Figure 3F), the overall study time is less than before.

### 3.5. The graduation-related situations and the further study or work plans of the students affected by the epidemic

#### 3.5.1. The impact of the COVID-19 epidemic on research topic

**FIGURE 4:**
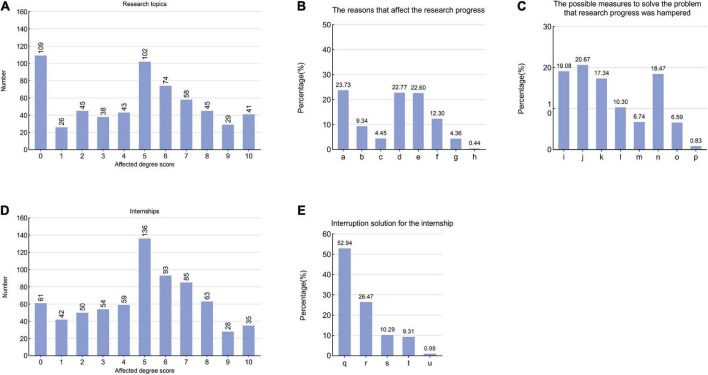
The impact of the COVID-19 epidemic on research topics and internships **(A)** The frequency distribution of the scores for the pandemic on research topics in the undergraduate and postgraduate students (0–10 points). **(B)** The reasons that affect the research progress. (a: Cannot enter the laboratory. b: There was a bad psychological state during the epidemic. c: There was physical discomfort during the epidemic. d: Without a learning environment at home, I cannot devote myself to research. e: During the epidemic, personal self-control decreased and laziness appeared. f: My mentor’s guidance and supervision decreased during the epidemic. g: Non-epidemic causes. h: Others). **(C)** The possible measures to solve the problem that research progress was hampered. (i: I hope the school will improve the scientific research equipment, establish a scientific research system and strengthen the scientific research management. j: I hope the school will extend laboratory opening hours while doing a good job of preventing and controlling the epidemic. k: I want to adjust the research design and reduce the difficulty of the project with the help of the instructor. l: I want to reach the conclusion directly based on the available research. m: I hope to improve the professional level of project instructors. n: I need to increase the time I invest in scientific research and improve the efficiency of related work. o: I hope my home will offer me a good learning environment. p: I am willing to abandon the subject). **(D)** The frequency distribution of the scores for the pandemic on internships in the undergraduate and postgraduate students (0–10 points). **(E)** Interruption solution for the internship. (q: I hope the school will offer other alternative internship programs to continue completing the internship. r: I hope the school will reduce our internship requirements. s: I hope the internship requirement will be eliminated. t: I am an independent internship unit, and the internship interruption has little impact on me. u: Others).

Of the 2,000 respondents, 30.50% had research topics in progress. The frequency distribution of research progress affected by the epidemic was presented in Figure 4A. The results suggested that graduates’ research topics were more affected by the epidemic than those of non-graduates (*P* = 0.001); however, there was no statistically significant difference in the impact degree of the epidemic on research topics between students of different genders, undergraduate students versus postgraduate students, and medical students versus non-medical students (all *P* > 0.05).

Among the eight reasons listed in the questionnaire that affected the research progress, a lack of access to the laboratory or research location (23.73%) and a lack of a family learning environment leading to an inability to focus on research (22.77%) were chosen as the main reasons. Other reasons for this and their response rates are shown in Figure 4B. To solve the problem that research progress was hampered during the epidemic, we gave the students some possible measures to choose from: 20.67% of students hoped that their school would extend laboratory hours while still doing a good job of preventing and controlling the epidemic; 19.08% of students hoped that their school would improve the scientific research equipment, establish a scientific research system and strengthen scientific management, and the selection of other measures were shown in Figure 4C.

#### 3.5.2. The impact of the COVID-19 epidemic on internship

In this study, 35.30% of the students had internship requirements at their current learning stage, and the frequency distribution of the scores representing the impact of the COVID-19 epidemic on their internships was shown in Figure 4D. Through analysis, we found that the internships of graduates were more affected by the epidemic than those of non-graduates (*P* = 0.003). In addition, there was no statistically significant difference in the degree to which the epidemic impacted internships between other demographic backgrounds (all *P* > 0.05).

In this study, 28.90% of students’ internships were interrupted because of the epidemic. Faced with this problem, 52.94% of students hoped that the school would offer alternative internship programs for completing their internship requirements, and 26.47% hoped that the school would reduce their internship requirements, other hopes could be found in Figure 4E.

#### 3.5.3. The impact of the COVID-19 epidemic on graduation and further study or work planning

**FIGURE 5:**
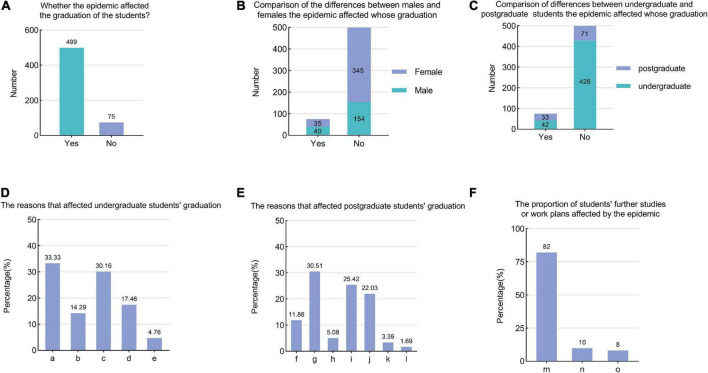
The impact of the COVID-19 epidemic on graduation and further study or work planning **(A)** Whether the epidemic affected the graduation of the students? **(B,C)** The chi-square test analysis results of the demographic background of the students affected by the epidemic at graduation. **(D)** The reasons that affected undergraduate students’ graduation. [a: Specialized courses did not meet the requirements of graduation credits (grade points). b: The requirements of graduation internships have not been completed. c: The participating research topics could not be concluded on time due to the impact of the epidemic. d: Did not complete the graduation project (thesis). e: Others]. **(E)** The reasons that affected postgraduate students’ graduation. [f: Failure to meet graduation credit requirements. g: Due to the impact of the epidemic, the graduation project was not completed within the specified time. h: Due to the influence of marriage and childbirth. i: Own behavior (such as less time invested in scientific research, unclear academic planning, low efficiency of scientific research output, etc.). j: Did not complete the graduation thesis. k: My tutor’s guidance was not of high quality. l: Others]. **(F)** The proportion of students’ further studies or work plans affected by the epidemic. (m: No effect. n: I had planned to continue my studies, but now I plan to look for a job directly after graduation. o: I had planned to look for a job directly after graduation, but now I intend to continue my studies).

This study also aimed to determine whether the graduation of the students was affected by the epidemic. Among the respondents, 574 undergraduate students or postgraduate students were scheduled to graduate at the end of the school year, but 13.07% of those students indicated that the COVID-19 epidemic may affect their graduation (Figure 5A). We found that the proportion of males and postgraduate students affected by the epidemic was higher than that of females and undergraduate students (Figures 5B, C). Among the students whose graduation was affected by the epidemic, a survey of undergraduate and postgraduate students about the factors that affected their graduation and the corresponding response rates were shown in Figures 5D, E.

Facing the problem that they may not be able to graduate on time, 32.95% of undergraduate students and 30.00% of postgraduate students hoped that the school would reduce their graduation requirements, 22.73% of undergraduate students hoped that their school would strengthen their guidance regarding graduation theses, and 20.00% of postgraduate students hoped that their mentors would strengthen their guidance and supervision. Other measures that students would like to receive can be found in Supplementary Text 4.

With regard to whether the COVID-19 epidemic had an impact on further studies or work plans of the students, our survey found that 82.00% of students reported no effects, 9.90% of students indicated that they had planned to continue their studies originally but now plan to look for jobs immediately after graduation, and 8.10% of students indicated that they originally intended to find a job directly after graduation but now plan to continue their studies (Figure 5F).

## 4. Discussion

Up to now, there is still a rebound in the epidemic in some parts of China, and students will continue to be affected by the epidemic under the “dynamic zero-COVID policy.” The findings of this study can provide some academic value for studying the impact of COVID-19 and other epidemics on students in China and other countries with the same severity of the epidemic.

This study found that the proportion of Chinese students whose mental health was affected was higher than that of students whose physical health was affected. A study found similar results: Americans reported more physical symptoms (including cough, fever, etc.), while Chinese reported more acute traumatic stress symptoms (14). Mental health is affected by social connections, online classes, and physical activity (15), and students with less physical exercise were significantly more stressed, while physical exercise may relieve stress (16). This study showed that, compared with pre-epidemic levels, the total amount of physical exercise in 48.05% of students decreased, and mental states in 21.90% of students were weakened. Whether there is a link between the two is not yet known, but improving physical exercise guidance for students during the pandemic may help them maintain good physical and mental health. In addition, this survey found that boredom and anxiety were the main mental health problems faced by Chinese undergraduate and postgraduate students during the pandemic, but 36.66% of students tended to self-adjust, and only 1.91% of students chose to seek help from a professional psychological counselor. We do not know why students rarely seek professional help, but we should provide them with platforms and services relevant to a student’s perspective, including sending emails and text messages to students to encourage them to communicate related issues (17).

One study found that younger groups (18–25 or 26–45) were most involved in social activities and were most negatively affected in terms of social and leisure aspects during the pandemic (18). In addition, one study showed that interpersonal communication has been severely affected by epidemic prevention and control measures (e.g., wearing masks) (19). In this study, non-medical students were more affected by the epidemic in their leisure and entertainment activities than medical students; non-medical students and graduates were more affected by the epidemic in their emotional life and interpersonal communication than medical students and non-graduates. This discrepancy is intriguing, and we need to explore the specific contributing factors further.

The level of emotional investment in online education is significantly lower than in traditional learning environments. This decline in emotional investment is mainly due to the decreased level of interaction between students or between students and teachers in the new learning environment (20). In this study, 25.75% of students indicated that they could not adapt to the change in learning styles, and 40.00% indicated that they preferred offline teaching. Furthermore, students’ study time showed an overall decreasing trend compared with that before the epidemic. Therefore, exploring sensible and effective forms of education in the context of the pandemic is an issue that needs to be addressed. This survey also found that the research progress of students was affected, it is particularly important to explore new methods to solve this problem. During the blockade of the epidemic, students in an institution use virtual laboratories to study more frequently and rely less on teachers, suggesting that virtual laboratories may play a prominent role in student education (21) and may be used as an online tool for students to practice during the pandemic.

In this study, 13.1% of the students expressed that the COVID-19 epidemic may affect their graduation. Among them, the proportion of males and postgraduate students affected was higher than those of females and undergraduate students, respectively. This result suggests that we should pay more attention to the difficulties encountered by males and postgraduate students in graduation. Faced with graduation problems, 20.00% of postgraduate students hoped that their mentors would improve their guidance and supervision. One university established a partnership with another university to help students who had already met their graduation requirements graduate early (22). Therefore, at both the school and teacher levels, we should be mindful of the academic pressures facing students and attempt to strengthen academic guidance to help them graduate without complications.

In conclusion, the results of this study show that COVID-19 has indeed affected the physical and mental health, daily life, and learning plan of students, and the impacts on different groups are inconsistent. We need to further explore its influencing factors and seek appropriate solutions, which will help students better cope with the impact of the epidemic.

## Data availability statement

The data analyzed in this study is subject to the following licenses/restrictions: In order to protect the privacy of the respondents, this study does not provide the original data, but the results of this study are based on these data for statistical analysis. Requests to access these datasets should be directed to LZ, 1280115391@qq.com.

## Ethics statement

This study was approved by the Ethics Committee of the Third Xiangya Hospital of Central South University. The patients/participants provided written informed consent to participate in this study.

## Author contributions

AG and LL designed the study. LZ collated the data, carried out the data analyses, and produced the initial draft of the manuscript. YYH contributed to the data collection. YZ, XL, HG, YBH, and SW contributed to the manuscript revision. All authors contributed to the article and approved the submitted version.
