# Supplementary material for: The impact of the COVID-19 pandemic on undergraduate and postgraduate students: A cross-sectional survey
Source: Front Psychiatry. 2023 Feb 3;14:1074597. doi: 10.3389/fpsyt.2023.1074597 (PMC9935598; doi:10.3389/fpsyt.2023.1074597)
Supplement: Supplementary file 1 [file Data_Sheet_1.docx]

Supporting Information

# Supplementary Texts

## Supplementary Text S1. The specific contents of the informed consent.

Hello, everyone:

The outbreak of COVID-19 has made our peaceful life unusual. Has your life, study or work been affected? Would you like to work with me to find solutions to these problems, so that students deeply affected by the epidemic can get help? We sincerely invite you to participate in the questionnaire survey on the impact of COVID-19 epidemic on undergraduate and postgraduate students in China conducted by the dermatology department of the Third Xiangya Hospital of Central South University. The purpose of this questionnaire is to understand the impact of COVID-19 epidemic on everyone's life, study and work, so as to explore measures to solve the problems faced by everyone during the epidemic. This questionnaire will not disclose any personally identifiable information of you, and all your responses will be kept confidential and kept anonymously. The data obtained from the survey are only used for academic exchange after sorting out. Please fill in according to your own real situation. It will take you 3-5 minutes to complete this questionnaire. Participation in this survey is based on voluntary principle. If you don't want to, you can refuse to participate. If you have any questions about your rights and interests, you can contact the Ethics Committee of the Third Xiangya Hospital of Central South University at 0731-88618938 during working hours.

## Supplementary Text S2. The results of the statistical analysis of the extent to which the physical and mental health of students in different risk-level areas were affected by the epidemic.

The risk level of the epidemic situation at the school location was different, and there was a statistical difference in the degree of students' physical health affected by the epidemic (*P*< .05). Further Bonferroni method showed that the physical health of students in medium-risk areas (Rank mean=1262.69) was more affected by the epidemic than those in low-risk areas (Rank mean =996.92) (*P.adj* = .031). However, there was no statistically significant difference in the degree of impact that the epidemic had on the mental health and the total amount of physical exercise of students in different risk-level areas (*P*> .05).

## Supplementary Text S3. The results of the statistical analysis of the extent to which the daily life of students in different risk-level areas were affected by the epidemic.

The risk level of the epidemic situation at the school location was different, and the emotional life of students affected by the epidemic was not exactly the same (*P*< .05). The Bonferroni test showed that the emotional life of students in high-risk areas (Rank mean =1219.50) was more affected by the epidemic than those in low-risk areas (Rank mean =995,58) (*P.adj* = .002). The Kruskal-Wallis H Test showed a statistically significant difference in the degree to which interpersonal communication was affected by the epidemic between students in different risk-level areas (*P*= .032), but after further pairwise comparisons, there was no statistically significant difference between the groups (*P*.adj > .05).

## Supplementary Text S4. Other measures that students would like to receive in the face of not being able to graduate on time.

Additionally, 20.45% of undergraduate students and 21.67% of postgraduate students believed that they needed to improve their basic theoretical knowledge and practical innovation ability, 12.50% of undergraduate students hoped that the school would improve the professional level of scientific research instructors, and 15.00% of postgraduate students hoped that the school would improve its annual evaluation of mentors to avoid retaining unqualified ones or indicated that the scientific research resources provided to students were limited. Moreover, 9.09% of undergraduate students and 13.11% of postgraduate students expressed that they could accept postponement of graduation.

# Supplementary Tables

## Supplementary Table S1. The Mann-Whitney U Test of the impact degree that the epidemic had on health status.

| Variables | | Physical health | | | Mental health | | | The total amount of physical exercise | | |
| --- | --- | --- | --- | --- | --- | --- | --- | --- | --- | --- |
|  |  | M (IQR) | *Z* | *P* | M (IQR) | *Z* | *P* | M (IQR) | *Z* | *P* |
| Gender | Male | 5.0 (5.0) | -2.606 | 0.009^*^ | 5.0 (4.0) | -1.291 | 0.197 ^ns^ | 5.0 (5.0) | -1.178 | 0.239 ^ns^ |
|  | Female | 5.0 (4.0) |  |  | 5.0 (3.0) |  |  | 5.0 (3.0) |  |  |
| Academic degree | Undergraduate | 5.0 (4.0) | -0.760 | 0.447 ^ns^ | 5.0 (3.0) | -0.580 | 0.562 ^ns^ | 5.0 (4.0) | -1.304 | 0.192 ^ns^ |
|  | Postgraduate | 5.0 (5.0) |  |  | 5.0 (4.0) |  |  | 5.0 (5.0) |  |  |
| Major | Medical | 3.0 (4.0) | -4.214 | 0.000^**^ | 4.0 (4.0) | -3.448 | 0.001^**^ | 4.5 (4.0) | -2.196 | 0.028^*^ |
|  | Non-medical | 5.0 (3.0) |  |  | 5.0 (3.0) |  |  | 5.0 (4.0) |  |  |
| Whether a graduate | Yes | 5.0 (4.0) | -3.122 | 0.002^*^ | 5.0 (4.0) | -4.244 | 0.000^**^ | 5.0 (3.0) | -2.101 | 0.036^*^ |
|  | No | 4.0 (5.0) |  |  | 5.0 (4.0) |  |  | 4.0 (4.8) |  |  |

Note: ns means *P*> .05, ***P*< .001, **P*< .05

## Supplementary Table S2. The Kruskal-Wallis H Test of variance of impact degree that the epidemic had on health status.

| Variables | | Physical health | | | | Mental health | | | | The total amount of physical exercise | | | |
| --- | --- | --- | --- | --- | --- | --- | --- | --- | --- | --- | --- | --- | --- |
|  |  | M (IQR) | *X^2^* | *P* | | M (IQR) | *X^2^* | *P* | | M (IQR) | *X^2^* | *P* | |
| Age (year) | 18-21 | 4.0 (4.0) | 5.309 | | 0.257 ^ns^ | 5.0 (3.0) | 4.607 | | 0.330 ^ns^ | 5.0 (3.0) | 12.848 | | 0.012^*^ |
|  | 22-25 | 5.0 (4.3) |  |  |  | 5.0 (5.0) |  |  |  | 5.0 (5.0) |  |  |  |
|  | 26-29 | 4.0 (5.0) |  |  |  | 5.0 (6.0) |  |  |  | 5.0 (4.0) |  |  |  |
|  | ≥30 | 3.0 (3.0) |  |  |  | 3.5 (4.0) |  |  |  | 4.0 (3.5) |  |  |  |
| The risk level of the epidemic at the school location | High-risk area | 2.0 (-) | 6.922 | | 0.031^*^ | 8.0 (-) | 5.656 | | 0.059 ^ns^ | 7.0 (-) | 3.706 | | 0.157 ^ns^ |
|  | Medium-risk area | 6.0 (3.5) |  |  |  | 5.0 (2.0) |  |  |  | 6.0 (2.5) |  |  |  |
|  | Low-risk area | 5.0 (4.0) |  |  |  | 5.0 (4.0) |  |  |  | 5.0 (4.0) |  |  |  |

Note: ns means *P*> .05, ***P*< .001, **P*< .05

## Supplementary Table S3. The Mann-Whitney U Test of the impact degree that the epidemic had on daily lives.

| Variables | | Leisure and entertainment activities | | | Emotional life | | | Interpersonal communication | | |
| --- | --- | --- | --- | --- | --- | --- | --- | --- | --- | --- |
|  |  | M (IQR) | *Z* | *P* | M (IQR) | *Z* | *P* | M (IQR) | *Z* | *P* |
| Gender | Male | 6.0 (4.0) | -0.621 | 0.535 ^ns^ | 5.0 (5.0) | -1.702 | 0.089 ^ns^ | 5.0 (4.0) | -0.769 | 0.442 ^ns^ |
|  | Female | 6.0 (3.0) |  |  | 5.0 (4.0) |  |  | 5.0 (4.0) |  |  |
| Academic degree | Undergraduate | 6.0 (4.0) | -1.022 | 0.307 ^ns^ | 5.0 (4.0) | -1.370 | 0.171 ^ns^ | 5.0 (4.0) | -1.235 | 0.217 ^ns^ |
|  | Postgraduate | 6.0 (3.5) |  |  | 5.0 (5.0) |  |  | 5.0 (4.0) |  |  |
| Major | Medical | 5.0 (4.0) | -3.186 | 0.001^*^ | 5.0 (4.3) | -2.409 | 0.016^*^ | 5.0 (4.0) | -4.329 | 0.000^**^ |
|  | Non-medical | 6.0 (3.0) |  |  | 5.0 (4.0) |  |  | 5.0 (3.0) |  |  |
| Whether a graduate | Yes | 6.0 (3.0) | -0.613 | 0.540 ^ns^ | 5.0 (4.0) | -3.613 | 0.000^**^ | 5.0 (4.0) | -2.994 | 0.003^*^ |
|  | No | 6.0 (4.8) |  |  | 5.0 (5.0) |  |  | 5.0 (3.8) |  |  |

Note: ns means *P*> .05, ***P*< .001, **P*< .05

## Supplementary Table S4. The Kruskal-Wallis H Test of variance of impact degree that the epidemic had on daily lives.

| Variables | | Leisure and entertainment activities | | | Emotional life | | | | Interpersonal communication | | | |
| --- | --- | --- | --- | --- | --- | --- | --- | --- | --- | --- | --- | --- |
|  |  | M (IQR) | *X^2^* | *P* | M (IQR) | *X^2^* | *P* | | M (IQR) | *X^2^* | *P* | |
| Age (year) | 18-21 | 5.0 (4.0) | 10.299 | 0.036^*^ | 5.0 (3.5) | 10.005 | | 0.040^*^ | 5.0 (4.0) | 7.079 | | 0.132 ^ns^ |
|  | 22-25 | 6.0 (3.0) |  |  | 5.0 (4.3) |  |  |  | 5.0 (4.0) |  |  |  |
|  | 26-29 | 7.0 (3.0) |  |  | 7.0 (3.0) |  |  |  | 7.0 (4.0) |  |  |  |
|  | ≥30 | 3.0 (4.3) |  |  | 3.5 (3.3) |  |  |  | 3.0 (2.3) |  |  |  |
| The risk level of the epidemic at the school location | High-risk area | 7.0 (-) | 4.650 | 0.098 ^ns^ | 8.0 (-) | 14.162 | | 0.001^*^ | 3.0 (-) | 6.883 | | 0.032^*^ |
|  | Medium-risk area | 6.0 (4.0) |  |  | 5.0 (2.0) |  |  |  | 6.0 (1.5) |  |  |  |
|  | Low-risk area | 6.0 (4.0) |  |  | 5.0 (4.8) |  |  |  | 5.0 (4.0) |  |  |  |

Note: ns means *P*> .05, ***P*< .001, **P*< .05
